# Sarcoma stratification by combined pH2AX and MAP17 (PDZK1IP1) levels for a better outcome on doxorubicin plus olaparib treatment

**DOI:** 10.1038/s41392-020-00246-z

**Published:** 2020-09-23

**Authors:** Marco Perez, José Manuel García-Heredia, Blanca Felipe-Abrio, Sandra Muñoz-Galván, Javier Martín-Broto, Amancio Carnero

**Affiliations:** 1 Instituto de Biomedicina de Sevilla (IBIS), Hospital Universitario Virgen del Rocío, Universidad de Sevilla, Consejo Superior de Investigaciones Científicas, Sevilla, Spain; 2 Ciber de Cancer, IS Carlos III, Madrid, Spain; 3 Departamento de Bioquímica Vegetal y Biología Molecular, Universidad de Sevilla, Sevilla, Spain; 4 Present Address: Departamento de Anatomía Patológica, Hospital Universitario Virgen del Rocío, Sevilla, Spain

**Keywords:** Sarcoma, Cancer models

## Abstract

Sarcomas constitute a rare heterogeneous group of tumors, including a wide variety of histological subtypes. Despite advances in our understanding of the pathophysiology of the disease, first-line sarcoma treatment options are still limited and new treatment approaches are needed. Histone H2AX phosphorylation is a sensitive marker for double strand breaks and has recently emerged as biomarker of DNA damage for new drug development. In this study, we explored the role of H2AX phosphorylation at Ser139 alone or in combination with MAP17 protein, an inducer of DNA damage through ROS increase, as prognostic biomarkers in sarcoma tumors. Next, we proposed doxorubicin and olaparib combination as potential therapeutic strategies against sarcomas displaying high level of both markers. We evaluate retrospectively the levels of pH2AX (Ser139) and MAP17 in a cohort of 69 patients with different sarcoma types and its relationship with clinical and pathological features. We found that the levels of pH2AX and MAP17 were related to clinical features and poor survival. Next, we pursued PARP1 inhibition with olaparib to potentiate the antitumor effect of DNA damaging effect of the DNA damaging agent doxorubicin to achieve an optimal synergy in sarcoma. We demonstrated that the combination of olaparib and doxorubicin was synergistic in vitro, inhibiting cell proliferation and enhancing pH2AX intranuclear accumulation, as a result of DNA damage. The synergism was corroborated in patient-derived xenografts (PDX) where the combination was effective in tumors with high levels of pH2AX and MAP17, suggesting that both biomarkers might potentially identify patients who better benefit from this combined therapy.

## Introduction

Sarcomas are rare tumors thought to be derived from mesenchymal precursor cells arising from bone, cartilage, or connective tissues, such as muscle, fat, peripheral nerves, fibrous, or related tissues.^1^ These malignancies comprise over 100 histological subtypes, being many molecular aberrations prevalent within specific sarcomas. Despite the existence of some cases of hereditary predisposition to sarcomas, like Li-Fraumeni Syndrome, the vast majority of these types of tumors appear sporadically.^2^ From genetic point of view, sarcomas have been traditionally classified into two broad categories, those with near-diploid karyotypes and simple genetic alterations, including translocations or specific activating mutations, and those with a complex and unbalanced karyotype.^3,4^ The mechanisms of development and progression of sarcoma remain elusive, and very few tumors are therapeutically targeted. Most of them have poor prognosis, due to late diagnosis when tumor is at advanced state.^5^ First-line sarcoma treatment options are still limited to traditional surgery, chemo, and radiotherapy. Furthermore, there are a few approved chemotherapeutic drugs for treating soft-tissue sarcoma, such as gemcitabin, eribulin, trabectedin, doxorubicin, ifosfamide, anthracyclines, and taxanes, although many of them show a high inefficiency rate and produce resistance cases.^6^ As a result, success in treatment of sarcomas remains extremely poor and requires a better understanding of pathogenesis for new treatment approaches.

Genes related to the response to DNA damage play essential roles in the maintenance of a healthy genome. Defects in cell cycle checkpoints and/or DNA repair genes, especially mutations or aberrant gene downregulation, are associated with a wide spectrum of human tumors.^7–9^ On the other hand, the probability of cancer development is higher due to the upregulation of both DNA damage response and repair genes, which usually generates higher resistance to DNA damaging therapies. Generally, high DNA damage levels correlates with a higher grade and worse patient outcomes.^8^ In addition, each tumor should be considered as an individual and specific disease, due to the presence of different alterations in the genome of the patient’s tumor. Thereby, the characterization of certain biomarkers capable of predicting cellular response to a specific treatment could improve survival of patients who meet those biomarkers requirements, avoiding the use of certain drugs with lower efficiency and high toxicity.

A commonly followed strategy to improve chemotherapeutic regimens is to increase DNA damage and impede its repair. In this sense, the use of Poly (ADP-ribose) polymerase 1 (PARP1) inhibitors (such as olaparib) has been broadly used, since PARP is a key initiator of the repair by recruiting the DNA repair machinery to the site of damage.^10,11^ However, despite its preclinical data, dose escalation studies in phase I showed high hematologic toxicities that hampered its clinical use and limited its possibilities.^12–21^ Therefore, a strong rationale is needed to combine PARP inhibitors with other first-line treatment, especially in sarcoma, to further advance in this area of tumor treatment. Stratifying patients by using biomarkers to predict a better outcome is a clear choice.

H2AX is a component of the histone octamer in nucleosomes that is phosphorylated in Ser139 (pH2AX) by kinases recognizing DNA damage, like DNA-dependent protein kinase (DNA-PK)^7^and Ataxia Telangiectasia Mutated.^22^ pH2AX detects DNA breaks therefore it helps to understand if a new drug causes DNA damage. With low levels of DNA damage, PARP1 promotes cell survival by repairing single strand breaks (SSBs) of DNA and preventing its progression to toxic double strand breaks (DSBs).^23^ If SSBs progress to DSBs, PARP1 induces H2AX phosphorylation, which induces BRCA1/2 recruitment to repair DSBs, which eventually prevents apoptotic cell death.^24^ As such, the presence and magnitude of pH2AX is an indicator of persistent, unrepaired DNA damage, being the phosphorylated histone studied as prognostic biomarker in early operable non-small cell lung cancer (NSCLC) and endometrial carcinomas.^25,26^ Drugs such doxorubicin or trabectedin are generally used to induce DNA damage and, subsequently, cell death, in different tumors or cells.^27–29^

On the other hand, MAP17 (PDZK1IP1, DD96, SPAP)^30–32^ is a small and non-glycosylated membrane protein located in the Golgi apparatus and plasma membrane usually deregulated in human carcinomas.^30,33,34^ Tumor cells overexpressing MAP17 show pro-oncogenic advantages^35–37^ related to an increase in cell dedifferentiation.^33,34,38^ This increased malignant behavior is associated with an increase of 30–40% in reactive oxygen species (ROS) induced by MAP17,^35,38,39^ moving the balance from low levels of DNA damage, that triggered repair and survival mechanisms, to high levels that can promote cell death, by inducing cellular or DNA damage beyond repair.^8,40^ Therefore, an increment in MAP17 levels can lead to increased DNA damage that can be detected using specific biomarkers, such as pH2AX. Both pH2AX and/or MAP17 have been characterized as biomarkers for some tumors.^33,41–44^

Here, we have characterized how both biomarkers can be used to predict the response of sarcoma tumors to a combined doxorubicin plus olaparib treatment. Our results in sarcomas, together with others previously obtained in other types of tumors, allow us to find a pattern that would allow the design of common strategies to treat different tumors.

## Results

### MAP17 is related with an increment in DNA damage in sarcoma

**Fig. 1:**
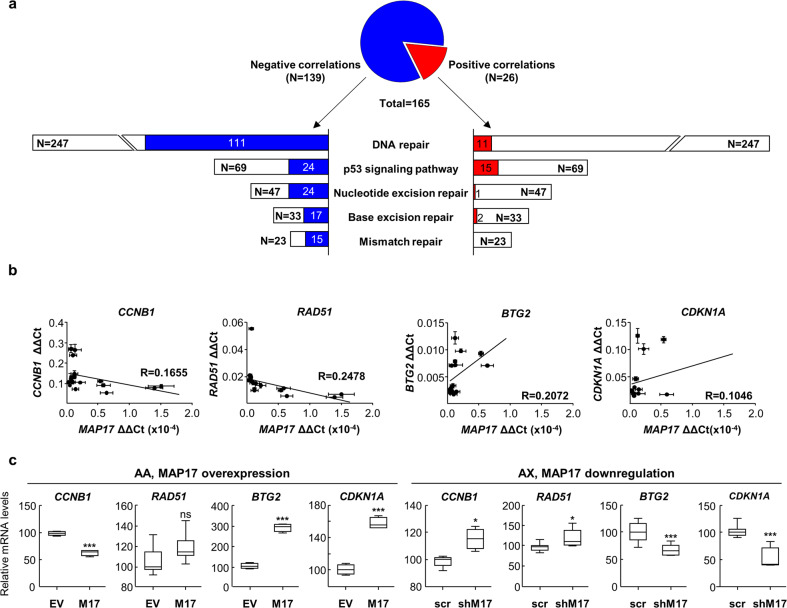
MAP17 correlates with DNA damage in sarcoma tumors. **a** MAP17 expression in sarcoma datasets correlates with genes related to DNA damage and repair. White bars represent the whole number of genes in each category, according to R2. Blue or red bars refer to the number of genes in each category correlated with *MAP17*. **b** Expression levels of genes negatively (*RAD51* and *CCNB1)* and positively (*BTG2* and *CDKN1A)* correlated with MAP17 in sarcoma cell lines showed a similar behavior to bioinformatics analysis. **c** *CCNB1*, *RAD51*, *BTG2*, and *CDKN1A* mRNA levels in control (EV) or overexpressing MAP17 (M17) AA cells or in scrambled shRNA (scr) or shRNA against MAP17 (shM17) AX cells. Student’s *t* test statistical analysis of the data was performed to find statistical differences (*p < 0.05; **p < 0.01; ***p < 0.001). Data are presented as the mean ± SD

To look whether DNA damage was one of the effects caused by MAP17 expression in sarcoma tumors, we looked for *MAP17* correlations with genes related to DNA damage using R2 software. To this end, we looked in the Gene Category “DNA repair” and in the KEGG pathway categories “p53 signaling pathway”, “Mismatch repair”, “Base excision repair”, and “Nucleotide excision repair” in sarcoma databases (Supplementary Data Table S1). We found a total of 139 and 26 genes related to DNA damage and repair (Supplementary Data Table S2), negatively or positively correlated with *MAP17* in at least a 25% of the databases considered (Fig. 1a). This list showed that MAP17 expression is mainly negatively correlated with processes related to DNA repair, which could be connected to the previous role of MAP17 as an inducer of ROS.^38,39,45^

From the list of identified genes, as in vivo control validation, we selected four genes; *RAD51* and *CCNB1* as genes negatively correlated to *MAP17*, and *BTG2* and *CDKN1A*, as genes positively correlated to *MAP17*. RAD51 is essential for homologous recombination, appearing in foci where it acts in repair reactions after DNA damage.^46^ In addition, the accumulation of CCNB1 after DNA damage caused by radiation induces apoptosis.^47^ *CDKN1A* encodes p21 protein which, like BTG2, is induced through p53 pathway after DNA damage.^48,49^ However, it is not yet known how MAP17 could affect the expression of these genes, although its interaction with NUMB^50^ could modify p53 pathway, due to the described interaction of NUMB with MDM2 that avoids the ubiquitination and degradation of p53.^51^ We analyzed the expression of these genes in a set of 11 sarcoma cell lines (AA, AX, BC, BG, CE, A673, CP0024, 93T449, Saos-2, HT-1080, and SW872), finding a similar correlation to the previously found in bioinformatics datasets (Fig. 1b). *RAD51* and *CCNB1* expression levels were lower in cells with higher MAP17 levels. On the other hand, *BTG2* and *CDKN1A* levels appeared increased in these cells with higher MAP17 levels. This suggests that, in sarcomas, *MAP17* expression could be correlated with DNA damage. Furthermore, we measured the expression level of these genes in two sarcoma cell lines with modified *MAP17* expression, previously described in our laboratory.^50^ Ectopically increased *MAP17* expression (in AA cell line), modified *CCNB1* mRNA levels with a significant decrease, while *BTG2* and *CDKN1A* mRNA levels increased. AX cell line, transfected with a shRNA against *MAP17*, showed similar behavior according to MAP17 levels. The reduction of MAP17 levels by the specific shRNA also caused the reduction of *BTG2* and *CDKN1A* mRNA levels, and a significant increase in *CCNB1* and *RAD51* mRNA levels (Fig. 1c). All these results pointed to a possible role of MAP17 as an inducer of DNA damage in sarcoma cells, so we decided to improve our knowledge using a previously described DNA damage biomarker, pH2AX.

### pH2AX levels in sarcoma

**Fig. 2:**
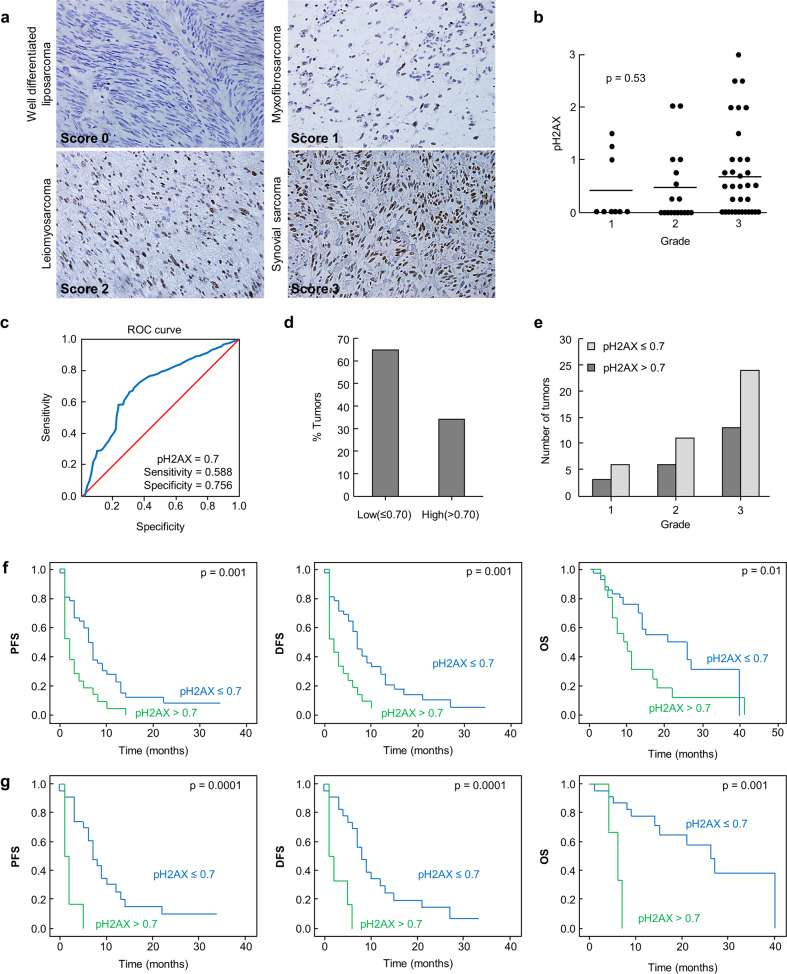
High pH2AX correlates with worse prognosis. **a** Representative pictures of pH2AX staining in different histological sarcoma samples. **b** pH2AX levels correlation with tumor grade in sarcoma showed that pH2AX levels are not connected with tumor grade in sarcoma. The mean values are represented for grade 1 (0.41), grade 2 (0.45) and grade 3 (0.65). ANOVA was performed to establish the statistical association between the pH2AX levels and grade of the tumor, without reaching a significant value lower than *p* < 0.05. **c** ROC curve for pH2AX showing sensitivity = 0.588 and specificity = 0.756 with the cut-off point of pH2AX > 0.7. **d** Overall distribution of pH2AX positive in sarcomas. Thirty-three percent showed higher staining levels and were considered positive for pH2AX. **e** Distribution of high/low pH2AX levels among tumor grade. **f** Progression-free survival (PFS), disease-free survival (DFS), and overall survival (OS) according pH2AX levels. **g** PFS, DFS, and OS according pH2AX levels in patients treated only with doxorubicin

In order to look for a possible connection between MAP17 and DNA damage in sarcoma tumors, we decided to focus in pH2AX, due to the previously described role of pH2AX as a functional marker of DNA damage.^42,44^ To this end, we analyzed 69 samples from a cohort of different types of sarcomas for which we had clinical information^52^ (Supplementary Data Table S3). Sarcoma tumors were divided into four scores, considering pH2AX staining (Fig. 2a). We found a similar distribution of pH2AX in sarcomas, regardless of the tumor grade (Fig. 2b) Using 0.7 as the pH2AX cut-off level as it depicted in the receiver operating characteristic (ROC) curve (sensitivity = 0.588, specificity = 0.756) (Fig. 2c), patients were divided in two groups, with high or low pH2AX levels. Of the 69 samples, 33% showed higher levels of pH2AX (Fig. 2d) and, according to the ROC curve, they were considered positive for pH2AX. These tumors appeared with the same ratio distribution between both groups among tumor stages (Fig. 2e). Due to these results that showed similar pH2AX levels at different tumor grades, we then analyzed patient survival considering pH2AX levels. Univariate Cox analysis showed that high pH2AX levels were associated with overall survival (OS), disease-free survival (DFS), and progression-free survival (PFS) (Table 1). Thereby, high pH2AX levels were predictive of worse PFS (*p* = 0.001), DFS (*p* = 0.001), and OS (*p* = 0.01) (Fig. 2f). Therefore, our data suggest that high levels of phosphorylated H2AX might be an independent marker of poorer prognosis in sarcoma patients. Treatment with doxorubicin, which induces DNA damage, showed a clear trend in DFS, PFS, and OS, according to pH2AX levels (Fig. 2g). This result showed that patients with low pH2AX levels showed a better prognosis when treated with doxorubicin. However, patient stratification according tumor grade was only significant for Grade 1 (Table 1). We also found by univariate Cox analysis, that both MAP17 and pH2AX are prognostic biomarkers in our cohort (Table 1). High pH2AX levels were significantly associated with worse DFS (*p* = 0.002), PFS (*p* = 0.006), and OS (*p* = 0.019). Higher MAP17 levels were only slightly associated with worse DFS (*p* = 0.047) and PFS (*p* = 0.05), with no relevance regarding OS. Based on these results, we analyzed both biomarkers in our univariate analysis in our cohort, with the combined expression designated as MAP17–pH2AX score (Table 1). Score 2 is referred to the presence of both biomarkers, while Score 0 is referred to their absence. Thereby, Score 2 appeared significantly associated with worse DFS (*p* = 0.003), PFS (*p* = 0.008), and OS (*p* = 0.009). For multivariate Cox analysis, the factors significantly associated with DFS, PFS, and OS were considered: pH2AX, MAP17, and differentiation. This analysis revealed that only pH2AX was a predictive marker of worse DFS, PFS, and OS (Table 2). Due to these results, we focus on pH2AX as a biomarker to improve the poor prognosis of these patients with high pH2AX and treated with doxorubicin.

**Table 1:** Univariate Cox proportional hazard regression analysis for OS, DFS and PFS

Characteristics	No.	OS HR (95% CI)	*p*	DFS HR (95% CI)	*p*	PFS HR (95% CI)	*p*
pH2AX (high vs. low)	21/63	2.220 (1.142–4.315)	0.019	2.457 (1.390–4.343)	0.002	2.195 (1.259–3.827)	0.006
MAP17 (high vs low)	25/65	1.470 (0.754–2.866)	0.258	1.719 (1.007–2.935)	0.047	1.692 (0.994–2.879)	0.05
Differentiation							
UD	2	1.0	0.023	1.0	0.127	1.0	0.127
Grade 1	10	0.062 (0.04–1.028)	0.052	0.260 (0.054–1.246)	0.030	0.171 (0.035–0.840)	0.030
Grade 2	18	0.537 (0.066–4.362)	0.561	0.566 (0.129–2.476)	0.165	0.345 (0.77–1.550)	0.165
Grade 3	35	1.077 (0.144–8.034)	0.943	0.495 (0.117–2.103)	0.120	0.311 (0.71–1.354)	0.120
MAP17–pH2AX Score 2 vs Score 0	12/42	3.123 (1.327–7.294)	0.009	3.072 (1.455–6.485)	0.003	2.711 (1.303–5.600)	0.008

Score 2: high levels of both pH2AX and MAP17; Score 0: low levels of both pH2AX and MAP17

*OS* overall survival, *DFS* disease-free survival, *PFS* progression-free survival, *HR* hazard ratio, *CI* confidence interval, *UD* undifferentiated, *MAP17–pH2AX* combined score for immunohistochemical expression of pH2AX and MAP17

**Table 2:** Multivariate Cox proportional hazard regression analysis for OS, DFS, and PFS

Characteristics	No.	OS HR (95% CI)	*p*	DFS HR (95% CI)	*p*	PFS HR (95% CI)	*p*
pH2AX (high vs low)	21/63	2.468 (1.244–4.896)	0.010	2.470 (1.351–4.516)	0.03	2.013 (1.091–3.712)	0.025
MAP17 (high vs low)	25/65			1.341 (0.765–2.351)	0.306	1.450 (0.817–2.573)	0.204
Differentiation							
UD	2	1.0	0.05	1.0	0.351	1.0	0.383
Grade 1	10	0.073 (0.04–1.275)	0.073	0.136 (0.013–1.404)	0.094	0.222 (0.038–1.296)	0.095
Grade 2	18	0.574 (0.071–4.663)	0.604	0.234 (0.028–1.964)	0.181	0.368 (0.079–1.721)	0.204
Grade 3	35	1.078 (0.144–8.079)	0.941	0.177 (0.021–1.460)	0.108	0.317 (0.070–1.437)	0.136

*OS* overall survival, *DFS* disease-free survival, *PFS* progression-free survival, *HR* hazard ratio, *CI* confidence interval, *UD* undifferentiated, *MAP17–pH2AX*: combined score for immunohistochemical expression of pH2AX and MAP17

### pH2AX determines sensitivity to combined treatment with doxorubicin plus olaparib in sarcoma cell lines

**Fig. 3:**
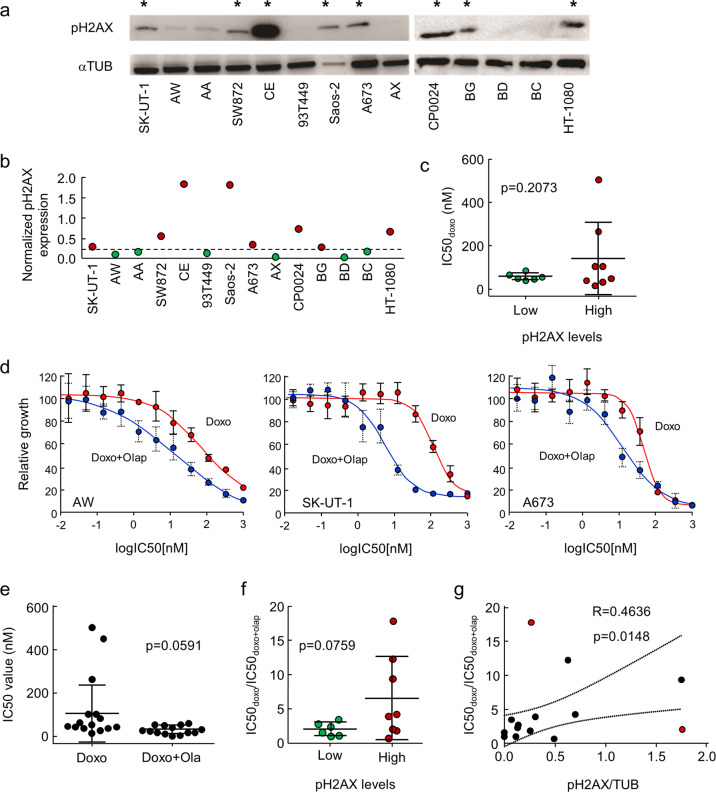
pH2AX levels determine sensitivity to combined doxorubicin plus olaparib treatment. **a** pH2AX levels in a panel of 14 sarcoma cancer cell lines. The cell lines were subdivided according to pH2AX levels, so that those with higher levels are marked with a (*). **b** Normalized (pH2AX/α-TUB) levels, according to a representative WB result. Dashed line separates cells with low or high pH2AX relative levels. **c** IC50 doxorubicin values according to low or high pH2AX relative levels. **d** IC50 curves for AW, SK-UT-1 and A673 sarcoma cell lines, treated with doxorubicin alone or a combined treatment of doxorubicin and a suboptimal dose of olaparib. **e** IC50 values for doxorubicin or combined doxorubicin plus olaparib treatments. **f** IC50_doxo_/IC50_doxo+olap_ of each sarcoma cell line tested, grouped by their relative pH2AX levels. Student’s *t* test statistical analysis of the data was performed to find statistical differences for IC50 (*p < 0.05; **p < 0.01; ***p < 0.001). Data are presented as the mean ± SD **g** Correlation of IC50_doxo_/IC50_doxo+olap_ and relative levels of pH2AX.95% CI are shown for linear regression

In order to analyze the correlation of pH2AX with sensitivity to doxorubicin, we used a panel of eight low-passage sarcoma cell lines generated directly from patient samples and six commercial cell lines of heterogeneous origin. Relative pH2AX levels allowed us to separate cells into a group with high levels (CE, Saos-2, CP0024, HT-1080, SW872, A673, SK-UT-1, BG) and other with low levels (AA, BC, 93T449, AW, AX, BD) (Fig. 3a, b). Then, we determined the IC50 value for doxorubicin in these cells (Supplementary Data Table S4), which allowed us to connect the sensitivity to doxorubicin to pH2AX levels, finding no differences in sensitivity due to pH2AX levels (Fig. 3c).

Next, we analyzed the effect on DNA repair inhibition by olaparib in combination with doxorubicin, and correlated this effect with pH2AX levels. To this end, we used a combined olaparib plus doxorubicin treatment. Olaparib concentration was set at a suboptimal dose, determined using its previously obtained IC50 values, to induce DNA damage without significantly killing cells (Supplementary Data Table S4). Interestingly, combined treatment of a suboptimal dose of olaparib with increasing levels of doxorubicin allowed us to observe that olaparib increases sensitivity to doxorubicin in all cell lines (Fig. 3d, Supplementary Data Table S4), regardless of the histological sarcoma tumor type (Fig. 3e). Analysis according to pH2AX levels showed a further reduction in IC50 values observed in cells with higher pH2AX by the combined olaparib plus doxorubicin treatment (Fig. 3f, Supplementary Data Table S4). Only for SW872, the sarcoma cell line with the lowest IC50 for doxorubicin, the combined treatment showed worse behavior, with a higher IC50 value. In this case, other unknown factors could be affecting the final results.

pH2AX levels are a readout of the levels of DNA damage in cells. Therefore, cells with increased pH2AX levels have higher DNA damage levels, turning more sensitive to agents that further increase DNA damage, such as olaparib and doxorubicin, which ultimately overcome the DNA repair ability of the cells and kill them. As consequence, the combined treatment causes a further drop in IC50 values in cells with higher pH2AX levels when doxorubicin is combined with olaparib treatment. Considering more than 85% of the sarcoma cells in our panel, we observed a moderate positive correlation (*R* = 0.4636) between the reduction in IC50 values and pH2AX levels (Fig. 3g). This suggests that the accumulation of DNA damage, measured as pH2AX levels in these cells, could determine the higher observed sensitivity between olaparib and doxorubicin.

### Combined treatment with olaparib and doxorubicin induces a higher stress rate according to pH2AX levels

**Fig. 4:**
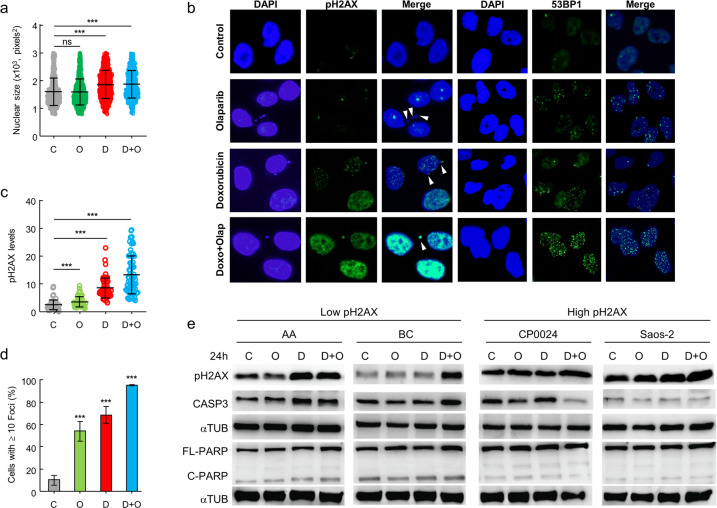
Olaparib increases DNA damage markers in sarcoma cell lines. **a** Analysis of variations in nuclear size of BC cells control or treated for 24 h with olaparib, doxorubicin, or combined treatment. **b** Analysis of pH2AX levels and 53BP1 foci after drug exposure. White arrows indicate the presence of micronuclei and nuclear buds, caused by each treatment. **c** Quantification of pH2AX. **d** Quantification of 53BP1 foci. **e** pH2AX, caspase-3, PARP (full length and cleaved) levels in two cell lines with low pH2AX levels (CP0024, Saos-2) and other two with high pH2AX levels (AA, BC). C control, O olaparib treatment, D doxorubicin treatment, D + O: doxorubicin plus olaparib treatment. Student’s *t* test statistical analysis of the data was performed to find statistical differences for IC50 (*p < 0.05; **p < 0.01; ***p < 0.001). Data are presented as the mean ± SD

In order to analyze the consequences of exposure to doxorubicin, olaparib or both drugs, we worked with several sarcoma cell lines with different pH2AX levels and different sensitivity to olaparib (Supplementary Data Table S4). BC cells were selected because of its high resistance to olaparib, so we exposed them to each treatment for 24 h. At first, we observed a significant increase in nuclear size due to treatment with doxorubicin (Fig. 4a). This result is compatible with a non-apoptotic cell death or an autophagic mechanism, as previously described for doxorubicin.^53,54^ Olaparib treatment, alone or in combination with doxorubicin, showed no effect on nuclear size. Each individual treatment caused the appearance of genotoxic stress signals such as micronuclei and nuclear buds^55^ (Fig. 4b), in a similar percentage. However, we found a clear increment in pH2AX levels due to doxorubicin treatment, appearing also in micronuclei, as previously described,^56^ while olaparib treatment produce only a slight increment in pH2AX levels (Fig. 4c). Nevertheless, the combined treatment produced a very significant increase in pH2AX levels, which also appeared in micronuclei. To confirm the effect induced by the combined therapy, we also measured the levels of 53BP1, which has been previously associated with an increment in DNA damage repair.^57^ We observed an increment in 53BP1 levels for each individual treatment, although the combination of doxorubicin with olaparib induced a greater number of nuclear foci containing the 53BP1 protein, concomitant with a strong pH2AX staining (Fig. 4b, d). This indicates that, although BC cells were resistant to olaparib, according to the IC50 value, this drug induces a level of DNA damage that can be bypassed by these cells.

To explore the effect induced by treatment, depending on the basal pH2AX levels, we selected two sarcoma cell lines with low (AA, BC) and other two with high (CP0024, Saos-2) levels of pH2AX (Fig. 3a). All four cell lines were treated with doxorubicin, olaparib, or the combined treatment for 24 h, looking for correlated changes in pH2AX, caspase-3, and PARP levels. PARP can be considered as a biomarker pointing to the type of cellular stress caused by doxorubicin treatment,^53^ due to the differences in its cleavage. Thereby, PARP cleavage by lysosomal proteases, typical of necrosis, results in a 55 kDa protein. However, proteolytic activation of pro-caspase-3, typical of the apoptotic program, leads to the active protease caspase-3,^58^ which cuts the 116-kDa form of PARP1 at the DEVD site in two small pieces, of 85 and 24 kDa.^59^ As consequence, the cleaved PARP is not able to repair DNA damage.^60^ We observed that pH2AX increases in all cells, regardless of the treatment, according to our results with pH2AX foci in BC cell line. In fact, we found very similar pH2AX levels both in WB and nuclear pH2AX quantification (Fig. 4c, e). The increment in pH2AX levels was higher in AA and BC cell lines, with lower basal pH2AX levels, while cells with higher pH2AX levels exhibited smaller increments (Fig. 4e). However, we found clearer differences in caspase-3 and, to a lesser extent, in cleaved PARP. Therefore, both AA and BC cell lines exhibited a significant increment in active caspase-3, which was reflected in an increment in cleaved 85 kDa PARP. However, CP0024 and Saos-2, with higher pH2AX levels, showed lower active caspase-3 and cleaved PARP levels. Both results suggest that genotoxic stress induced by doxorubicin, olaparib and combined treatment is not equally activated in cells.

### Combined low MAP17 plus low pH2AX levels are predictors of a better prognosis in sarcoma

**Fig. 5:**
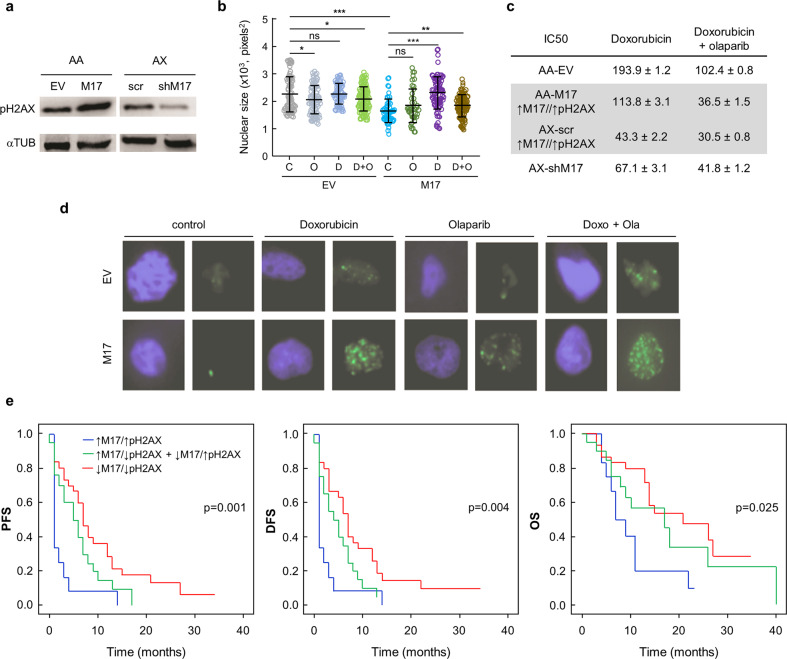
MAP17 overexpression increase DNA damage markers in sarcoma cell lines. **a** pH2AX levels in AA and AX sarcoma cell lines with modified MAP17 expression. **b** Nuclear size in cell line AA, control or overexpressing MAP17, after each treatment. Student’s *t* test statistical analysis of the data was performed to find statistical differences for IC50 (*p < 0.05; **p < 0.01; ***p < 0.001). Data are presented as the mean ± SD **c** IC50 in AA or AX cell lines with modified MAP17 transcriptional levels, treated with doxorubicin alone or combined treatment of doxorubicin plus olaparib. **d** Representative 53BP1 foci in AA EV or M17 cell lines, after treatment for 24 h with olaparib, doxorubicin, or both treatments. **e** Correlation of PFS, DFS, and OS with pH2AX and MAP17 levels measured as a dichotomous variable: MAP17 and pH2AX high rates (blue), low rates (red), or low/high or high/low rates (green)

Therefore, as suggested by the previously found correlation between MAP17, ROS, and DNA damage,^35,38,39^ we used the two cell lines with modified MAP17 levels.^50^ AX, from the group of cells with higher MAP17 levels, had MAP17 expression downregulated by an shRNA. In the AA sarcoma cell line, we increased its expression by ectopic expression of MAP17 cDNA. To elucidate whether MAP17 overexpression was related to increased DNA damage, we measured pH2AX levels in both cell lines. As a result, we found that increased MAP17 expression was correlated with an increment in pH2AX levels, indicating a correlation between the two markers (Fig. 5a). As for BC cells, we measured nuclear size, which were smaller in cells overexpressing MAP17 regarding to control cells, suggesting other changes in nuclear structure due to MAP17 overexpression (Fig. 5b). In addition, each treatment produced small changes in nuclear size of control cells, while changes were more significant in MAP17 overexpressing cells, suggesting an increased sensitivity to the treatments. Therefore, we treated both cell lines, with different MAP17 levels, to obtain the IC50 values for doxorubicin alone or the combined treatment with olaparib. As a result, higher MAP17 levels induced increased sensitivity to the combined therapy (Fig. 5c). We also found that the ectopic expression of MAP17 increases 53BP1 levels, regardless of the treatment (Fig. 5d). Like for BC cells, each of the treatments produced an increment in nuclear structures related to genomic instability, finding no differences due to MAP17 overexpression. These results points to a functional connection between MAP17 expression and DNA damage.

Altogether, these results suggest that the combined pH2AX and MAP17 high levels could be considered as biomarkers to predict cell sensitivity to certain drugs increasing DNA damage. Thus, we reanalyzed the same cohort of 69 patients, taking into account not only pH2AX but also MAP17 levels, measured previously by immunohistochemistry.^41^ Using 0.7 as the cut-off for pH2AX levels, and a cut-off point of 0.75 for MAP17 levels, as previously described,^41^ we found, similar to our previous results showed in Fig. 2f, that higher levels of both pH2AX and MAP17 were predictive of worse PFS (*p* = 0.001), DFS (*p* = 0.004), and OS (*p* = 0.025) (Fig. 5d). However, patients with low levels of both pH2AX and MAP17 showed a better prognosis, while high levels of only one of the biomarkers showed an intermediate prognosis. These data, in combination with the in vitro reduction of IC50 due to the combined doxorubicin and olaparib treatment, suggest that patients with a worse prognosis could show a better response to drugs increasing DNA damage if they present both high levels of pH2AX and MAP17.

### Sarcoma PDXs with high levels of pH2AX and MAP17 are more sensitive to doxorubicin plus olaparib

**Fig. 6:**
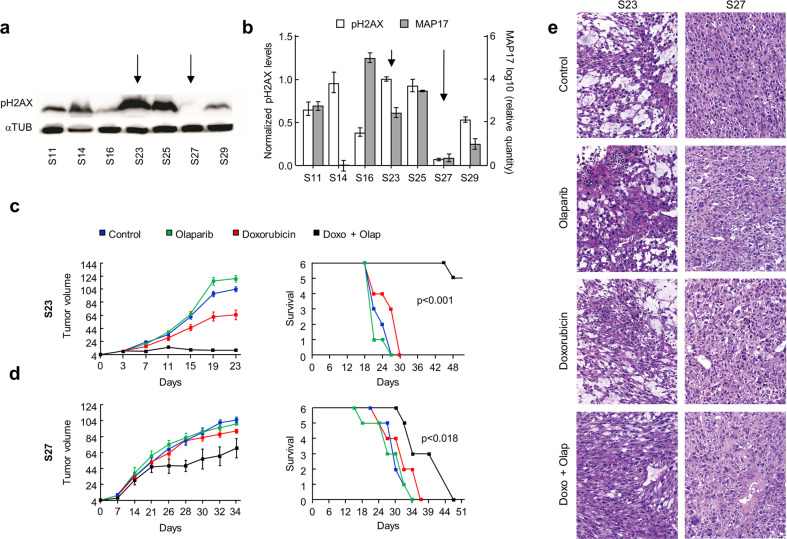
Effect of doxorubicin, olaparib or combined treatment in sarcoma tumors in vivo. **a** pH2AX levels in each sarcoma PDX model. **b** Relative pH2AX levels and transcriptional levels of MAP17 in each sarcoma PDX model. Data are presented as the mean ± SD. **c**, **d** Tumor response to doxorubicin, olaparib or combined treatment in S23 (high pH2AX/MAP17 levels) and S27 (low pH2AX/MAP17 levels), measured by changes in tumor volume. The left graph shows the average ± SD of tumor growth. The right graph shows the survival of the mouse cohorts euthanized by humane endpoint when the tumor reaches 1 cm^3^. ANOVA test was performed to find statistical differences. **e** Hematoxylin and eosin staining of S23 and S27 PDXs derived tumors

Finally, we decided to study whether MAP17 and pH2AX behave not only as biomarkers but are causal modifiers of the response to combined therapy. Thus, we tested how sarcoma patient-derived xenografts (PDXs) respond, in vivo, to doxorubicin, olaparib, or combined treatment. From a panel of cells derived from sarcoma PDXs,^61^ we selected S23 and S27 models, both undifferentiated pleomorphic sarcoma. The S23 tumor showed higher pH2AX levels (Fig. 6a, b) and also higher transcriptional MAP17 levels (Fig. 6b), than the S27 tumor. Therefore, we selected one model, S23, predicted as worse prognosis, and the other, S27, predicted as better prognosis, according to pH2AX and MAP17 levels. S23 or S27 tumors were engrafted subcutaneously in 24 mice for each model and grown until tumors reached a volume of 50 mm^3^. The mice were then randomly distributed for the treatment with doxorubicin, olaparib, combined treatment, or solvent alone (PBS). As a first result, mice with non-treated tumors of S23 model, with high pH2AX and MAP17 levels, died earlier than those with tumors of S27 model (Fig. 6c). S23 tumors treated with doxorubicin showed a small decrease in their volume (not shown), although survival was not affected. However, treatment of these tumors with doxorubicin plus olaparib caused arrest of tumor growth. The tumor disappeared from the flank of five out of six mice treated with doxorubicin plus olaparib, and all these mice survived. However, mice treated with either doxorubicin or olaparib individually showed similar results to control mice, without improving survival. The PDX model S27, with lower pH2AX and MAP17 levels, showed only a slight response to combined doxorubicin plus olaparib treatment (Fig. 6d). In fact, S27-derived tumors responded by slightly delaying tumor growth with only a slight improvement in survival. Again, mice treated with either doxorubicin or olaparib alone showed similar survival to control mice. At the end of the experiment, one of the mice harboring each tumor subtype, treated with solvent or any of the treatments considered, was euthanized and the histology of their tissues was analyzed. We found no differences in PDX tumors generated from S23 or S27 due to each treatment (Fig. 6e). Therefore, mice with tumors derived from sarcoma S23, with a poor prognosis and high pH2AX and MAP17 levels, showed better survival rates than mice with tumors derived from sarcoma S27 after treatment with olaparib plus doxorubicin. These results are in line with those obtained from both the cohort analysis and those obtained from cell cultures.

## Discussion

Here, we showed that the levels of biomarkers pH2AX and MAP17, individually or in combination, can be used to predict the response of sarcoma tumors to drug treatment of doxorubicin plus olaparib.

MAP17 is a protein whose expression, in normal conditions, is limited only to kidney proximal tubule cells,^62,63^ increasing their expression during tumor progression.^4,64^ MAP17 could also be considered as a biomarker in tumors, since its high levels are associated with an increment in endogenous ROS,^33,34,38^ which induce DNA damage and DDR signaling.^8^ MAP17 correlations in sarcoma datasets (Fig. 1) allowed us to find a negative correlation between its expression and processes connected to DNA repair, suggesting a possible functional connection between MAP17 and DNA damage repair. Furthermore, MAP17 ectopic expression induces increased sensitivity to the treatment with certain ROS-inducing drugs.^41,42,45^ Nevertheless, higher MAP17 levels, in sarcoma patients, are prognostic of poor survival.^41^ The seemingly opposite properties of poor survival and increased sensitivity to certain treatments of MAP17 turn this protein into a suitable biomarker to improve the treatment and survival of certain patients with a specific molecular signature. Thereby, in colorectal cancer, patients with high levels of both MAP17 and pH2AX showed better OS and DFS after radiotherapy and olaparib treatment,^42^ suggesting that both biomarkers should be analyzed together for the design of a personalized treatment.^42,44,62^ Likewise, the data extracted from our cohort showed that sarcomas with high pH2AX levels have a poor prognosis, regardless of the tumor origin. Furthermore, analysis of patient survival in our cohort showed a poorer prognosis when both MAP17 and pH2AX levels were highly expressed, showing that both biomarkers could be used to stratify patients with poorer prognosis for specific treatments that could improve their survival.

It is interesting, however, that high MAP17 levels strongly correlates with inflammation in other tumor types.^63,64^ Therefore, the immune system might influence also the poor prognosis of these tumors, through pro-tumorigenic activity. In this case, it will be interesting to analyze whether this inflammation-derived effect actively contributes to the poor prognosis observed in MAP17 positive tumors.

The poor prognosis for doxorubicin-treated tumors could be turned to a better prognosis when doxorubicin is combined with olaparib treatment, as we determined in our PDXs models (Fig. 6). We demonstrate that the treatment of cells overexpressing MAP17 with doxorubicin and the DNA repair inhibitor olaparib caused increased sensitivity compared to control cells. These data suggest that the increased sensitivity is due to an accumulation of non-repaired DNA damage. Furthermore, combined treatment of PDX sarcoma tumors with doxorubicin plus olaparib showed a differential response related to MAP17 and pH2AX levels. These results are in agreement with those behind the synergy between PARP inhibitors and trabectedin, BRCA1/2 status, or PARP1 expression in cells,^11,42,65–67^ and fueled the idea that increasing DNA damage along with the inability to repair it will be a good therapeutic option. In line with this, a doxorubicin^28^ and trabectedin resistant leiomyosarcoma cell line restored its sensitivity to these DNA damage agents by adding PARP inhibitors.^66^ Also, in osteosarcoma cells, the doxorubicin plus olaparib combined treatment incremented the apoptotic rate.^68^ Furthermore, recent clinical results have shown that combining trabectedin with olaparib allowed a partial response in sarcoma patients.^27^

These results are in the direction of personalized treatments according to the expression of each biomarker. Therefore, both biomarkers could be used to determine the combined use of olaparib and doxorubicin in sarcoma tumors not responding to conventional therapy. This could provide a new approach to treat sarcoma tumors, with only a few defined treatments. It should be interesting to know how sarcoma tumors with high levels of both biomarkers respond to other sarcoma first-line drugs, like ifosfamide.

Furthermore, we showed that sarcoma cells induced pH2AX according to their basal levels after drug treatment. Thus, cells with higher pH2AX values showed a small increment in its expression after drug treatment. These results suggest that in cells with worse prognosis there are higher rates of DNA damage that can be compensated by an active DNA repair system. However, it has been postulated that MAP17-induced ROS levels may contribute to reaching a threshold by further increasing ROS by anticancer drugs that can induce cell death.^38^ Therefore, the higher MAP17 expression allowed that, with lower pH2AX increments, cell death could be induced. Our results suggest a mechanism independent of caspase-3 activation. There are different pathways to trigger programmed cell death, being one of them caspase-3 independent. This mechanism has been related to inflammation and NFAT translocation to nuclei.^69,70^ MAP17 has also been connected to an increment in nuclear NFAT2 levels.^63,64^ Although more research is needed, it could be possible that higher MAP17 levels could induce increased sensitivity through a caspase-3 independent apoptotic program. Furthermore, higher pH2AX levels have also been detected in inflammatory processes,^71,72^ suggesting that both high pH2AX and MAP17 levels could be biomarkers in both ROS/DNA damage and inflammatory diseases. More research is needed to connect the causality of both processes in the evolution and drug sensitivity of tumors.

Because ROS is a potent proapoptotic insult, inducing DNA damage measured as an increase in pH2AX levels, we hypothesize that a further increase in MAP17 might sensitize to therapies that increase DNA damage switching the balance toward apoptosis, especially in tumors that showed a high level of damage beforehand. The detection of general behaviors in different tumor types could allow designing treatments for a specific model, more referred to the expression of certain proteins instead of a tumor type. Thus, and according to previous data,^42,44^ the combination of MAP17 and pH2AX could be considered as potential combined biomarkers for the design of specific strategies allowing better responses in patients.

## Material and methods

### Bioinformatics analysis

In order to find *MAP17*-correlated genes, we selected 11 databases for sarcoma tumors (see Supplementary Data Table S1). All databases are freely accessible through the R2 website (R2: Genomics Analysis and Visualization Platform, http://r2.amc.nl). We used five different gene filters: “DNA damage”, from Gene Category, and the KEGG pathway categories “p53 signaling pathway”, “Mismatch repair”, “Base excision repair”, and “Nucleotide excision repair”, options included in R2. Correlations were searched using the *MAP17* probes listed in Supplementary Data Table S1, establishing a *p* value lower than 0.05 to identify significant differences. From the list of correlated genes, we separated positively *MAP17-*correlated genes from negatively *MAP17-*correlated genes, generating two gene lists for each database (Supplementary Data Table S2).

### Human sarcoma cell lines and culture conditions

The primary sarcoma cell lines (AA, AX, AW, BC, BD, BG, CE, and CP0024) used in this study were previously characterized.^29,41,61,73^ A673, Saos-2, SK-UT-1, 93T449, HT-1080, and SW872 were commercial sarcoma cell lines. AA, AW, BC, BD, BG, and CE cells were maintained as a subconfluent monolayer in F-10 medium (Sigma). AX, Saos-2, SK-UT-1, 93T449, HT-1080, and SW872 were cultured using DMEM (Sigma), while A673 and CP0024 were maintained in RPMI (Sigma). Other characteristics of each cell line appear in Supplementary Data Table S5. All media were supplemented with 10% FBS, penicillin–streptomycin antibiotics (Sigma) and Fungizone (Amphotericin B, Sigma) as previously described.^29,41,61,73^ All cell lines were authenticated and regularly tested for mycoplasma. AA cell line, transfected to overexpress MAP17, and AX cell line, transfected with a shRNA against MAP17, were previously described^50^ and maintained in complete DMEM media supplemented with puromycin 0.5 μg ml^−1^.

### Quantitative mRNA determination

Total RNA was purified and retrotranscribed to cDNA as previously described.^74^ The expression of *MAP17* (Hs00906696_m1), *CCNB1* (Hs01030099_m1), *RAD51* (Hs00947967_m1), *BTG2* (Hs00198887_m1), and *CDKN1A* (Hs00355782_m1) was determined using an ABI 7900HT PCR system (Applied Biosystems). qPCR reactions were performed in 384-well plates using TaqMan Gene Expression Assays (Applied Biosystems). *GAPDH* (Hs03929097_g1) expression was used as an internal control. The relative amounts of mRNA were expressed as 2^−ΔΔCt^. Relative mRNA quantification and statistical analysis of qPCR data were performed using RQ Manager 1.2.1 software (Applied Biosystems).

### Tumor samples for immunohistochemical studies

The cohort of 69 patients for immunohistochemical studies and the correlation of clinico-pathological features were obtained from the Sarcoma Research Spanish Group Trial 20, Geis.^52^ The cohort of patients with clinical follow-up is described in Supplementary Data Table S3.^52^ Briefly, there were an equitable distribution by gender, men being 53% and the other 47% women. Nevertheless, there was a wide distribution by age, from 20 to 72 years. The most represented types of sarcomas were leiomyosarcoma (*n* = 22, 31.9%), liposarcoma (*n* = 13, 18.8%), and undifferentiated pleomorphic sarcoma (*n* = 12, 17.4%). Other sarcomas appeared in minor proportion, like fibrosarcoma (*n* = 5, 7.2%), hemangiopericytoma (*n* = 3, 3.4%), synovial sarcoma (*n* = 3, 4.3%), neurogenic sarcoma (*n* = 3, 4.3%), fibromyxoid sarcoma (*n* = 1, 1.5%), angiosarcoma (*n* = 1, 1.5%), and mesenchymal sarcoma (*n* = 1, 1.5%). Up to 76% of the tumors were metastatic at the time of diagnosis. For the study, all patients gave their written informed consent according to a protocol approved by the local ethics committee (CEI 2013/PI002). All tissue samples and patient information were treated in accordance with the Declaration of Helsinki.

### Immunohistochemistry

Three-micrometer slice sections was obtained from TMA blocks (collected from surgery at diagnostic and prior to treatment) and applied to coated, immunochemical slides (DAKO, Glostrup, Denmark). The slides were baked overnight in an oven at 56 °C, deparaffinized in xylene for 20 min, rehydrated through a graded ethanol series and washed with PBS. A heat-induced epitope retrieval step was performed by heating a slide in a sodium citrate buffer solution at pH 6.5 for 2 min in a conventional pressure cooker. After heating, the slides were incubated with proteinase K for 10 min and rinsed in cold running water for 5 min. Endogenous peroxide activity was quenched with 1.5% hydrogen peroxide (DAKO) in methanol for 10 min, and incubated with anti-MAP17 (1:4)^30,34,35,37^ and anti-pH2AX (Ser139) (ab11174 from Abcam) antibodies for other 40 min. After incubation, immunodetection was performed with the EnVision (DAKO, Glostrup, Denmark) visualization system using diaminobenzidine chromogen as substrate, according to the manufacturer’s instructions. Immunostaining was performed on a TechMate 500 automatic immunostaining device (DAKO) and measured by a double-blind visual assessment using microscope according to the anatomopathological experience of pathologists. Sample scoring was performed by semiquantitative microscopic analysis, considering the signal intensity, evaluated by the sum of the staining intensity scores and the staining area. We used the score obtained by the intensity levels, assigning level 0 (absence), 1 (weak), 2 (medium), and 3 (strong). To evaluate pH2AX staining, two pathologists experienced in sarcoma, evaluated independently the representative histological sections of the tumor, distinguishing inflammatory cell infiltrating the tumor and stroma for assigning the Score described in “Materials and methods”. The thresholds used were the score of 0.7 for pH2AX and 0.75 for MAP17,^41^ obtained by ROC curve, as the most relevant.

### Cytotoxicity assay

Doxorubicin was freshly prepared as a 10 μM stock solution in sterilized deionized water for each experiment. Olaparib was prepared as a 1 mM stock solution in DMSO. Both drugs were used independently to determine IC50 values. Cell lines were seeded in 96-well plates (5000–10,000 cells per well, depending on cell size). At 24 h after seeding, treatment was applied for 96 h. Cell proliferation was determined by the MTT assay and confirmed by crystal violet staining. For the combined treatment, we added to each cell line decreasing levels of doxorubicin and a suboptimal dose of olaparib, described in Supplementary Data Table S5. The IC50 was calculated using GraphPad Prism software.

### Tumor samples for PDX generation

Tumor tissues were obtained from the surgical resection of sarcomas carried out at Virgen del Rocio University Hospital (Seville, Spain). All patients gave their written informed consent according to a protocol approved by the local ethics committee (CEI 2013/PI002). The experiments were performed according to the European guidelines for laboratory animal care. This study was approved by the IBIS Institutional Animal Care and Use Committee.

### PDX generation

Sarcoma tissue samples from a single tumor area were obtained and preserved in F-10 medium (Sigma) containing 10% fetal bovine serum, penicillin, streptomycin, and amphotericin B (100 mg/ml each; Sigma). The samples were kept for less than 2 h in cell culture medium at room temperature before implantation. Each tissue was divided into two parts. One part was frozen, and the remaining part was cut into small 2–3-mm-diameter fragments to be used for subcutaneous implantation into 6-week-old Foxn1^nu^ athymic nude female mice (Harlan Laboratories, Netherlands). Upon reaching a size of 1500 mm^3^, the mice were euthanized, and the tumors were re-grown similarly to perform the indicated experiments.

### In vivo treatments

To initiate the experiments, each sample was xenografted in mice. Once the tumors reached 1.5 cm^3^, they were harvested, cut into 2 × 2 × 2-mm blocks and implanted. Experiments were performed using cohorts of six animals for each group. Mice were randomly allocated to the olaparib, doxorubicin, doxorubicin plus olaparib and control (solvent only) groups. Once the tumor grew to 20 mm^3^, the mice were treated for 3 weeks twice per week (doxorubicin) or three per week (olaparib). Mice cohorts were treated with olaparib (50 mg/kg body weight, 3 × week, in 0.9% NaCl), doxorubicin (1.5 mg/kg body weight, 2 × week in 0.9% NaCl), both treatments or with saline serum (0.9% NaCl). Mice were monitored daily looking for distress signs and weighed twice a week, finding no signs of toxicity. Tumor size was measured using a caliper according to the following equation: tumor volume = [length × width^2^]/2. The experiments were terminated when the tumor reached 1 cm^3^.

### Western blot analysis

Western blot analysis was performed as previously described.^41^ Briefly, cells were washed twice with PBS and lysed by sonication in RIPA buffer as previously described.^74^ Samples were separated on 6–15% gradient SDS-PAGE gels (BioRad), transferred to nitrocellulose membranes (Protran BA83; Whatman) and immunostained. The following primary antibodies and dilutions were used: anti-pH2AX (Ser139) (1:2000; Cell Signaling, 9718S), anti-Caspase-3 (E8) (1:500, Santa Cruz, sc7272), anti-PARP (46D11) (1:1000; Cell Signaling, 9532S), and anti-α-tubulin (1:5000; Sigma, 9026). Horseradish peroxidase-labeled rabbit anti-mouse (1:3000, Amersham, ab97046) and goat anti-rabbit (1:3000, Abcam, ab6721) secondary antibodies were used.

### Immunostaining of 53BP1 foci and pH2AX

BC cells were seeded on glass coverslips and cultured for 16 h. Then, 10 nM doxorubicin, 100 μM olaparib, or 10 nM doxorubicin plus 30 μM olaparib for combined treatment was added. After 24 h, the coverslips were fixed in 4% paraformaldehyde for 5 min at room temperature, washed twice with PBS, permeabilized with 0.5% Triton X-100 in PBS for 5 min and washed twice with PBS. Samples were incubated in blocking solution (PBS plus 3% bovine serum albumin) at 37 °C for 1 h, followed by incubation for 2 h at room temperature with an anti-53BP1 antibody (Novus Biologicals, NB100-304) diluted 1:100 or anti-pH2AX (Ser139) (Cell Signaling, 9718 S) diluted 1:400. After washing with PBS, cells were incubated with a species-specific Alexa 488-conjugated secondary antibody diluted 1:500 in blocking buffer for 1 h at room temperature in the dark. The nuclei were counterstained with DAPI, and the slides were mounted using Prolong Gold Antifade reagent (Life Technologies). The samples were visualized under an inverted System microscope (Olympus IX-71-ZDC). The mean fluorescence intensity was measured for a minimum of 300 cells per condition using Olympus imaging software. The plotted values represent the means of each condition. Furthermore, nuclear size was determined, measuring at least 50 cells, using ImageJ software.

### Statistical analysis and definitions

The Kaplan–Meier method was used for survival analysis, using the Cox proportional hazards model to adjust the explanatory variables, obtain the *p* values and estimate the HR. Multivariate logistic regression was used to obtain odds ratios and confidence intervals (95% CI). Pearson’s correlation measured the dependency between the quantitative variables. ROC curve analysis was performed to evaluate MAP17 and pH2AX cut-off points. Furthermore, the log-rank test was used to compare the survival distributions between high and low MAP17 levels. Statistical calculations were performed using SPSS 22.0 software. Survival analysis was performed using univariate and multivariate Cox regression hazard analysis and survival curves derived from Kaplan–Meier survival analysis. PFS was defined as the survival time (in months) of a cancer patient without worsening of their disease. DFS was defined as the time, after primary cancer treatment, that patients survive without any signs of cancer relapse. OS was defined as the time (in months) from the date of diagnosis to the date of the last medical record.

For mRNA expression levels and determination of IC50 values, three independent experiments were performed, and Student’s *t* test was performed to determine significant differences (*p* < 0.05). In order to detect significant differences in nuclear size, Student’s *t* test was performed.

The ANOVA test was used to analyze differences in pH2AX levels in sarcoma tumors according to their grade, and in PDXs tumors.

## Supplementary information

Supplementary Information

## Data Availability

The datasets supporting the results of this article are included in Supplementary Table 1 and are freely accessible through R2 (Genomics Analysis and Visualization Platform, http://r2.amc.nl). Also, data from patient cohort included in Supplementary Table 4 are freely accessible in ClinicalTrials.gov.
