# Supplementary material for: Sarcoma stratification by combined pH2AX and MAP17 (PDZK1IP1) levels for a better outcome on doxorubicin plus olaparib treatment
Source: Signal Transduct Target Ther. 2020 Sep 23;5:195. doi: 10.1038/s41392-020-00246-z (PMC7508862; doi:10.1038/s41392-020-00246-z)
Supplement: Supplementary file 1 — Supplementary Information [file 41392_2020_246_MOESM1_ESM.docx]

Supplementary Materials for

**Sarcoma stratification by combined pH2AX and MAP17 (PDZK1IP1) levels for a better outcome on doxorubicin plus olaparib treatment.**

Marco Perez^1,2^, José Manuel García-Heredia^1,2,3^, Blanca Felipe-Abrio^1,2^, Sandra Muñoz-Galván^1,2^, Javier Martín-Broto^1^, Amancio Carnero^1,2^*.

Correspondence to: acarnero-ibis@us.es

**This PDF file includes:**

Tables S1 to S5

| **Table S1**. List of sarcoma datasets used in this work | | |
| --- | --- | --- |
| Dataset | MAP17 probe | Sarcoma |
| Heiskanen (GSE92689) | 219630_at | Tumor Alveolar Rhabdomyosarcoma |
| Dirksen (GSE63157) | 2411175 | Tumor Ewing Sarcoma |
| Delattre (GSE7007) | 219630_at | Tumor Ewing Sarcoma |
| Davicioni (Pubmed link: 16849537) | 219630_at | Tumor Rhabdomyosarcoma |
| Skapek (GSE114621) | 2411175 | Tumor Rhabdomyosarcoma |
| TCGA (ID: SARC) | PDZK1IP1_10158 | Tumor Sarcoma |
| Schafer Welle | 219630_at | Mixed Rhabdoymyosarcoma |
| Kuijjer (GSE42352) | ILMN_1708580 | Mixed Osteosarcoma (Mesenchymal) |
| Aqeilan (Pubmed link: 22350417) | 219630_at | Mixed Osteosarcoma |
| Savola (GSE17679) | 219630_at | Mixed Ewing Sarcoma |
| Lawlor (GSE68776) | 2411176 | Mixed Ewing Sarcoma |

| **Table S2**. List of identified genes correlated with MAP17 in, at least, 25% of the analyzed datasets | | | | |
| --- | --- | --- | --- | --- |
| Negative correlations | | | | Positive correlations |
| *ABL1* | *EI24* | *PCNA* | *SHPRH* | *BBC3* |
| *ALKBH1* | *EPC2* | *PMS1* | *SMC1A* | *BTG2* |
| *ALKBH2* | *ERCC1* | *PMS2* | *SMC3* | *CCNB3* |
| *APAF1* | *ERCC3* | *PMS2CL* | *SSRP1* | *CCND3* |
| *APEX1* | *ERCC8* | *PMS2L2* | *SUMO1* | *CD82* |
| *ASF1A* | *EXO1* | *POLB* | *SUPT16H* | *CDKN1A* |
| *ATR* | *FANCB* | *POLD2* | *TDG* | *CIB1* |
| *BCCIP* | *FANCC* | *POLE* | *TDP1* | *ELN* |
| *BID* | *FANCG* | *POLE2* | *TNFRSF10B* | *ERCC6* |
| *BLM* | *FANCL* | *POLE3* | *TOP2A* | *FAS* |
| *BRCA1* | *FANCM* | *POLG2* | *TOPBP1* | *GADD45B* |
| *BRCA2* | *FBXO18* | *POLH* | *TP53* | *MBD4* |
| *CCNB1* | *GORAB* | *PRKDC* | *TYMS* | *MDM2* |
| *CCNB2* | *GTF2H1* | *PRPF19* | *UBE2N* | *MSH4* |
| *CCND1* | *GTF2H3* | *PTTG1* | *UBE2V2* | *PARP3* |
| *CCND2* | *GTF2H5* | *RAD18* | *UHRF1* | *PERP* |
| *CCNE2* | *H2AFX* | *RAD21* | *UNG* | *PML* |
| *CCNG2* | *HMGB1* | *RAD51* | *USP1* | *RPRM* |
| *CDK1* | *HUS1* | *RAD51AP1* | *XPA* | *SERPINB5* |
| *CDK4* | *LIG1* | *RAD51C* | *XRCC1* | *SESN1* |
| *CDK7* | *MDC1* | *RAD52* | *XRCC4* | *SFN* |
| *CDKN2A* | *MDM4* | *RAD54L* | *XRCC5* | *THBS1* |
| *CETN2* | *MLH3* | *RBM14* | *XRCC6BP1* | *TNP1* |
| *CHAF1A* | *MNAT1* | *RECQL* | *FEN1* | *TP53AIP1* |
| *CHAF1B* | *MRE11A* | *RECQL4* | *HMGB2* | *UBE2B* |
| *CHEK1* | *MSH2* | *RFC1* | *CCNE1* | *XRCC2* |
| *CHEK2* | *MSH6* | *RFC2* | *RFC4* |  |
| *CRY1* | *NCOA6* | *RFC3* | *BRIP1* |  |
| *CSNK1E* | *NEIL3* | *RFWD2* | *CYCS* |  |
| *CUL4B* | *NONO* | *RPA1* | *FANCF* |  |
| *DCLRE1A* | *NSMCE1* | *RPA2* | *MLH1* |  |
| *DCLRE1B* | *NSMCE2* | *RPA3* | *PARP4* |  |
| *DCLRE1C* | *NUDT1* | *RRM2* | *POLI* |  |
| *DDB2* | *OGG1* | *RUVBL2* | *UBE2A* |  |
| *EEF1E1* | *PARP1* | *SFPQ* |  |  |

| **Table S3.** Clinical characteristics of the patients (N=69). | | |
| --- | --- | --- |
|  | Number of patients | Percentage |
| Gender |  |  |
| Men | 37 | 53.6 |
| Women | 32 | 46.4 |
| Histology |  |  |
| Liposarcoma | 13 | 18.8 |
| Undifferentiated Pleomorphic Sarcoma | 12 | 17.4 |
| Hemangiopericytoma | 3 | 4.3 |
| Leiomyosarcoma | 22 | 31.9 |
| Synovial Sarcoma | 3 | 4.3 |
| Neurogenic Sarcoma | 3 | 4.3 |
| Fibrosarcoma | 5 | 7.2 |
| Unclassified sarcoma | 4 | 5.8 |
| Fibromyxoid Sarcoma | 1 | 1.5 |
| Angiosarcoma | 1 | 1.5 |
| Chondrosarcoma | 1 | 1.5 |
| Other | 1 | 1.5 |
| Staging |  |  |
| Locally advanced | 16 | 23.2 |
| Metastatic | 53 | 76.8 |
| Treatment |  |  |
| Doxorubicin 75 mg m^-2^ 3w x 6 cycles | 35 | 50.7 |
| Trabectedin 1.1 mg m^-2^ + Doxorubicin 60 mg m^-2^, 3w x 6 cycles | 34 | 49.3 |

**Reference**

1. Martin-Broto, J. *et al.* Randomized Phase II Study of Trabectedin and Doxorubicin Compared With Doxorubicin Alone as First-Line Treatment in Patients With Advanced Soft Tissue Sarcomas: A Spanish Group for Research on Sarcoma Study. *Journal of Clinical Oncology* **34**, 2294-2302 (2016).

| **Table S4.** Relative MAP17 and pH2AX levels and IC50 values of sarcoma cell lines used in this study. | | | | | | |
| --- | --- | --- | --- | --- | --- | --- |
| Cell line | MAP17  (mRNA level) | pH2AX^Ser139^ | IC_50_ Doxorubicin (nM) | IC_50_ Olaparib (μM) | [Olaparib] (μM) for combined treatment | IC_50_ Doxorubicin (nM) + Olaparib |
| SK-UT-1 | 5.58532x10^-5^ | 0.107938766 | 105.3 ± 11.6 | 9.4 ± 0.6 | 3.7 | 5.9 ± 0.4 |
| AW | 1.1437x10^-4^ | 0.085517989 | 66.6 ± 4.7 | 88.9 ± 2.7 | 33.4 | 19.3 ± 1.8 |
| AA | 6.21882x10^-5^ | 0.034766044 | 84.7 ± 4.5 | 113 ± 2.0 | 33.4 | 31.2 ± 1.5 |
| SW872 | 2.62208x10^-4^ | 0.111330573 | 16.7 ± 1.6 | 6.6 ± 0.2 | 3.7 | 25.7 ± 0.8 |
| CE | 4.55244x10^-5^ | 1.87008937 | 504.3 ± 50 | 83.2 ± 3.2 | 33.4 | 54 ± 1.3 |
| 93T449 | 1.32199x10^-4^ | 0.012140753 | 40.5 ± 7.8 | 5.9 ± 1.0 | 3.7 | 42.2 ± 1.2 |
| Saos-2 | 1.30192x10^-5^ | 0.607485454 | 40.6 ± 2 | 31.1 ± 6.3 | 11.1 | 20.3 ± 5.1 |
| A673 | 2.72762x10^-5^ | 0.08464252 | 48.4 ± 3.5 | 27.2 ± 0.3 | 11.1 | 12.6 ± 2.3 |
| AX | 5.34323x10^-4^ | 0.046965051 | 46.9 ± 5.3 | 87.9 ± 4.7 | 33.4 | 30.6 ± 8.1 |
| CP0024 | 2.84148x10^-5^ | 0.135240297 | 267 ± 8.8 | 9.0 ± 0.4 | 3.3 | 63.4 ± 7.4 |
| BG | 7.64036x10^-5^ | 0.083284157 | 104.4 ± 2.3 | 49.4 ± 2.8 | 33.4 | 58 ± 4.3 |
| BD | 1.11012x10^-4^ | 0.010655401 | 56 ± 1.8 | 114.8 ± 13.9 | 33.4 | 55.7 ± 8.3 |
| BC | 2.5699x10^-4^ | 0.017131038 | 47.5 ± 1.0 | >300 | 30 | 20.9 ± 3.8 |
| HT-1080 | 1.68955x10^-5^ | 0.142560996 | 30.9 ± 3.9 | 37.3 ± 1.2 | 11.1 | 2.5 ± 0.1 |

| **Table S5.** Sarcoma cell lines used in this study. | | | | |
| --- | --- | --- | --- | --- |
| Cell line | Tumor of Origin | Phenotype | Doubling time | Culture conditions |
| SK-UT-1 | Uterine Leiomyosarcoma | Epithelial | 24 h | DMEM |
| AW | Myxoid Liposarcoma | Fibroblastic | 48 h | F10 |
| AA | Leiomyosarcoma | Epithelial | 36 h | F10 |
| SW872 | Liposarcoma | Fibroblastic | 24 h | DMEM |
| CE | Rhabdomyosarcoma | Fibroblastic | 48 h | F10 |
| 93T449 | Retroperineal Liposarcoma | Fibroblastic | 72 h | DMEM |
| Saos-2 | Osteosarcoma | Epithelial | 48 h | DMEM |
| A673 | Ewing Sarcoma | Fibroblastic | 24 h | RPMI |
| AX | Myxoid Liposarcoma | Epithelial | 48 h | DMEM |
| CP0024 | Leiomyosarcoma | NA | 36 h | RPMI |
| BG | Myxoid Fibrosarcoma | Fibroblastic | 24 h | F10 |
| BD | Ewing Sarcoma | NA | 96 h | F10 |
| BC | MPNST | Epithelial | 96 h | F10 |
| HT-1080 | Fibrosarcoma | Epithelial | 36 h | DMEM |
| NA: Not Available | | | | |
